# High Resistance and Biofilm Tolerance to Antimicrobials of Marine Bacteria From Brazilian Deep‐Sea Sediment

**DOI:** 10.1111/1758-2229.70364

**Published:** 2026-05-19

**Authors:** Amanda Simão Dias, Sílvia Dias de Oliveira, Renata Medina‐Silva

**Affiliations:** ^1^ Imunology and Microbiology Laboratory, School of Health and Live Sciences Pontifical Catholic University of Rio Grande do Sul, PUCRS Porto Alegre Brazil; ^2^ Geobiology Group, School of Health and Live Sciences Pontifical Catholic University of Rio Grande do Sul, PUCRS Porto Alegre Brazil

**Keywords:** antimicrobials, biofilm tolerance, deep‐sea bacteria, microbial resistance, One Health

## Abstract

Antimicrobial resistance studies have focused on clinical bacteria, neglecting the role of resistant isolates in natural environments. However, oceans are daily contaminated with high loads of antimicrobials and resistant bacteria from agro‐industrial and urban activities. Deep‐sea sediment is a challenging environment that may select microbial strains with resistance to chemicals and ability to form biofilms, becoming a potential reservoir of resistance genes. We evaluated the susceptibility to antimicrobials of six *Pseudomonas* sp., five *Bacillus* sp., two *Brevibacillus* sp. and two *Paenibacillus* sp. from deep‐sea sediments of the Pelotas Basin (Brazil) by the disk diffusion and microdilution tests. *Pseudomonas* and Bacillales were tested against 11 and 7 antimicrobials, respectively. Biofilms of susceptible isolates were exposed to antimicrobials to determine the minimum biofilm inhibitory concentration (MBIC) and the minimum biofilm eradication concentration (MBEC). All *Pseudomonas* were resistant to aztreonam at very high concentrations (up to 2048 μg/mL). MBIC values were significantly higher than respective MICs, and only one third of biofilms were eradicated. These results underscore the importance of the study, as one of the first reporting antimicrobial tolerance of biofilms of cultivable bacteria from deep‐sea sediments, contributing to the knowledge of bacterial resistance in these environments, concerning One Health issues.

## Graphical abstract

Antimicrobial resistance and biofilm‐specific tolerance of cultivable *Pseudomonas*, *Bacillus*, *Brevibacillus* and *Paenibacillus* isolated from deep‐sea sediments of a methane‐seep area off the Southern Brazilian coast. These findings underscore the role of deep‐sea environments as potential reservoirs for antimicrobial‐resistant bacteria (ARB) and resistance genes (ARG), aligning with the One Health framework.

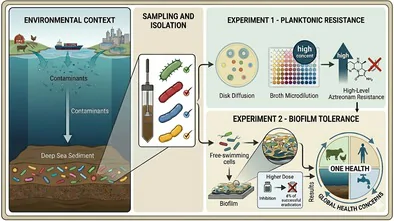

## 1 Introduction

Marine environments harbour a large portion of the planet's microorganisms, as more than 70% of the Earth's surface is covered by oceans (Glöckner et al. 2012). These species are present in the most diverse forms (Glöckner et al. 2012) and play different key roles in structuring and balancing marine communities and ecosystems (Munn 2019; Laiolo et al. 2024).

Despite the importance of the oceans in maintaining Earth's biosphere balance, these systems face numerous disturbances caused by human activities. Several contaminants reach oceanic waters every year, coming from industry, plastic and microplastic waste, pesticides and antimicrobials from different origins (Jambeck et al. 2015; Duleba et al. 2018; Ma et al. 2021). These xenobiotics can impact marine ecosystems and lead to consequences not only at the local level but also globally, ultimately affecting human populations (Chen et al. 2019; Habibi et al. 2022).

Antimicrobial resistance (AMR) is currently seen as a global public health problem (World Health Organization (WHO) 2023). Despite this recognition, most studies focus mainly on resistant microorganisms isolated from clinical origin, while only recently microbial isolates from natural environments have been studied in the context of AMR (Suzuki et al. 2017; Sweileh and Moh'd Mansour 2020). The rampant use of antimicrobial drugs across several sectors has accelerated the selection of resistant microorganisms (Holmes et al. 2016; Zhuang et al. 2021; World Health Organization (WHO) 2023). Although the substantial load of antimicrobials released into the oceans is diluted (Larsson and Flach 2021), chronic exposure to subinhibitory concentrations can drive adaptation, gene expression changes and dissemination of resistance to these agents for different bacterial species in these environments (Kohanski et al. 2010; Hatsoy and Martiny 2015; Christaki et al. 2020; Xu et al. 2023). Therefore, AMR can no longer be treated as a problem found only in healthcare facilities, as natural environments can be major contributors to the acquisition and spread of resistance genes (Hernando‐Amado et al. 2019).

The detection of antimicrobial resistance genes (ARGs) in bacteria from marine environments has increased, indicating their growing prevalence in these ecosystems (Li et al. 2020; Makkaew et al. 2021; Castaño‐Ortiz et al. 2023). In this context, marine environments are estimated to serve as important reservoirs for such genes (Marti et al. 2014). Moreover, beaches and the marine coast in general may represent important potential foci of resistant microorganisms, as these are areas of interface between ocean ecosystems and the human population (Dang et al. 2008a; Maravić et al. 2012; Yang et al. 2013; Luczkiewicz et al. 2015; Carney et al. 2019; Cuadrat et al. 2020). The prevalence of genes encoding resistance to tetracyclines, sulfonamides, β‐lactams and quinolones has already been observed in coastal areas (Dang et al. 2008b; Jang et al. 2018). Many of these genes are located on plasmids, potentially facilitating their efficient dissemination among diverse marine microorganisms through horizontal gene transfer, primarily via bacterial conjugation or transformation (Yang et al. 2013; Cuadrat et al. 2020).

Surface and coastal systems are connected to deep ocean areas through oceanic currents and biological pump processes. This makes deep‐sea sites potential final sinks and long‐term reservoirs for organic particles containing contaminants (Wang et al. 2025), as well as microbes (Chen et al. 2024). Moreover, deep‐sea sediment can be a challenging environment for microorganisms, as it presents high‐pressure levels, low oxygen concentrations and an intense flow of gases, selecting strains adapted to such conditions (Duleba et al. 2018; Ketzer et al. 2020). Among such adaptations, the resistance to chemicals and the ability to form tolerant biofilms have been highlighted in the literature (Yang et al. 2013; Chen et al. 2019; Habibi et al. 2022). In this context, deep‐sea sediments can act as potential reservoirs of ARGs across the oceans. Nevertheless, while numerous studies have investigated AMR in coastal and pelagic zones, research focusing on resistant bacteria from deep‐sea sediments remains limited.

Evaluating antimicrobial susceptibility in microorganisms from marine environments aligns with the One Health concept, emphasizing the ecological nature of this issue and its repercussions for the health of both human and nonhuman animals, as well as the overall environment (McEwa and Collignon 2018). Therefore, this study is one of the few aimed at profiling the AMR and biofilm‐specific tolerance of cultivable bacteria from the genera *Pseudomonas*, *Bacillus*, *Brevibacillus* and *Paenibacillus* recovered from the unique deep‐sea sediment environment of a methane‐seep submarine fan area located off the Southern Brazilian coast.

## 2 Experimental Procedures

### 2.1 Origin of Bacterial Isolates

**FIGURE 1:**
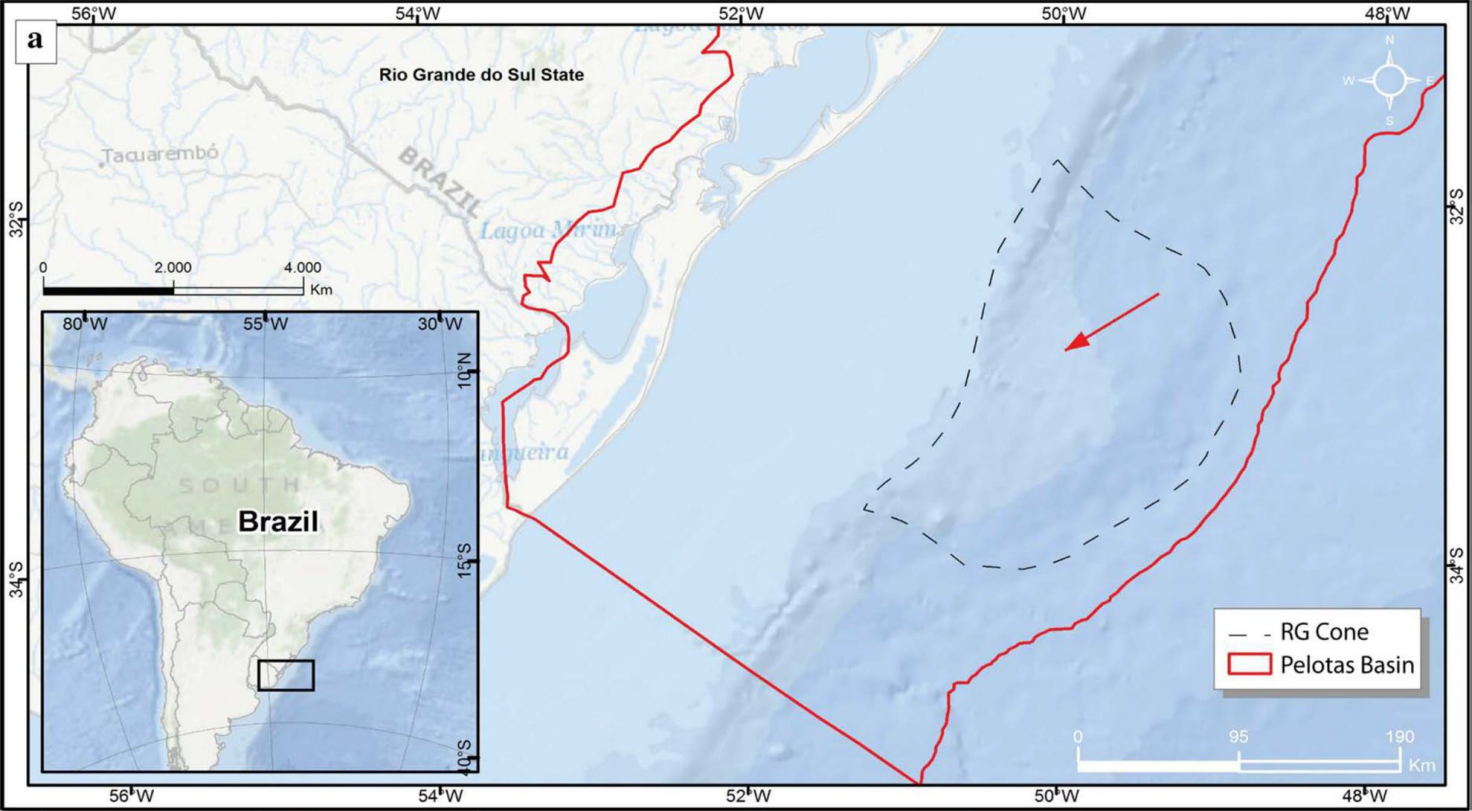
Map of the Rio Grande Cone location, within the Pelotas Basin in the Southwestern Atlantic, Brazil (modified from Medina‐Silva et al. 2018), the sampling site of deep‐sea sediment bacteria used in this study.

Sediment samples were previously collected within the Rio Grande Cone (RGC), a submarine fan described as a methane seepage site located at the Pelotas Basin (Brazil), in the Southwestern portion of the Atlantic Ocean (Miller et al. 2015), during two oceanographic campaigns MR11 (2011) and MD195 (2013), using piston corers (Figure 1). Bacterial strains were immediately isolated from sediment samples taken from depths of 0–3 m below the seabed at sites with water depths of 1800 to 2500 m. The media Brain Heart Infusion (BHI), yeast extract‐peptone‐dextrose (YPD) or nitrate mineral salts medium (NMS) with methane (simulating the original environment) were used for isolation and storing of the bacterial strains at −80°C with dimethyl sulfoxide (DMSO) or glycerol in the proportion of 5% or 20% (v/v), respectively. Bacillales constituted the most abundant bacterial taxon cultured from these deep‐sea sediment samples, whereas isolates of the genus *Pseudomonas* were exclusively recovered using NMS medium under a methane‐enriched atmosphere (Proenca et al. 2022). Consequently, to ensure a representative sampling of the two primary taxonomic groups identified at this site, 15 isolates were selected for the present study. Among these, six had been previously identified via 16S rRNA gene sequencing as *Pseudomonas* sp., while nine belonged to the order Bacillales, encompassing the genera *Bacillus* (*n* = 5), *Brevibacillus* (*n* = 2) and *Paenibacillus* (*n* = 2) (Table 1) (Proenca et al. 2022). For some experiments, *Pseudomonas aeruginosa* ATCC 27853 and *Bacillus cereus* ATCC 33019 strains were used as references.

**TABLE 1:** Taxonomic identification of isolates used in this study, previously performed by Proenca et al. (2022).

Bacterial group	Isolates	Identification
*Pseudomonas* genus	MD330.6	*Pseudomonas* sp.
	MD330.9	*Pseudomonas* sp.
	MD330.10	*Pseudomonas* sp.
	MD330.11	*Pseudomonas* sp.
	MD332.06	*Pseudomonas* sp.
	MD332.08	*Pseudomonas* sp.
Bacillales order	MET16	*Paenibacillus* sp.
	MET17	*Paenibacillus* sp.
	MR6	*Brevibacillus* sp.
	MR16	*Brevibacillus* sp.
	MR29	*Bacillus* sp.
	MR38	*Bacillus* sp.
	MR39	*Bacillus* sp.
	MR46	*Bacillus* sp.
	MR53	*Bacillus* sp.

### 2.2 Antimicrobial Susceptibility Tests in Planktonic Condition

The antimicrobial susceptibility of the isolates was evaluated by disk diffusion and broth microdilution tests. The protocols and antimicrobials used in both susceptibility tests were based on the criteria of the European Committee on Antimicrobial Susceptibility Testing (EUCAST) and the Clinical & Laboratory Standards Institute (CLSI) for isolates belonging to the genus *Pseudomonas* and to genera of the Bacillales order. As the marine isolates did not grow properly at 35°C (the recommended temperature for these tests), the incubation temperature used in both tests was 25°C (for 18–24 h), in which these isolates present their optimal growth. The precision of minimum inhibitory concentration (MIC) data sets has already been evaluated under different incubation conditions and demonstrated not to be affected in the temperature range of 22°C–35°C (Smith et al. 2018). In the disk diffusion test, a decrease in precision has been previously observed under incubation temperatures lower than 35°C and incubation times over 24 h (particularly 22°C for 48 h), which was not related to differences in the mean diameter zone sizes (Smith and Kronvall 2015; Smith et al. 2018). Despite this eventual limitation, other studies already used the same incubation time and temperature as we employed for both the disk diffusion test (Miller et al. 2003; Wamala et al. 2018) and the broth microdilution test (Smith et al. 2018) when assessing the susceptibility of environmental isolates to antimicrobials.

#### 2.2.1 Kirby Bauer—Disk Diffusion Test

*Pseudomonas* isolates were tested against 11 antimicrobials from six different classes: ceftazidime, cefepime, gentamicin, tobramycin, amikacin, aztreonam, ciprofloxacin, levofloxacin, imipenem, meropenem and piperacillin‐tazobactam, while Bacillales isolates were submitted to seven drugs from five distinct classes: ciprofloxacin, levofloxacin, imipenem, meropenem, erythromycin, clindamycin and linezolid. A suspension of the isolates was prepared in sterile saline (0.85% NaCl) to a turbidity compatible with 0.5 McFarland scale (~1 × 10^8^ CFU/mL), then inoculated using a sterile swab onto cation‐adjusted Mueller–Hinton plates (CAMH), where antimicrobial disks were added. The plates of marine isolates were incubated at 25°C and those of the reference strains at 37°C, for 18–24 h. Afterward, the size of each inhibition zone was measured, and the results were compared and classified according to the CLSI and EUCAST guidelines.

#### 2.2.2 Broth Microdilution Test

The MIC was determined by broth microdilution, using 96‐well plates. The isolates that proved to be resistant in the disk diffusion test were exposed to different concentrations of the antimicrobial agents to which they presented such a profile, in triplicates. The aim was to evaluate the minimum antimicrobial concentration required to inhibit these resistant isolates, since this can vary among bacteria as a result of distinct genotypic and/or phenotypic features they may exhibit against antimicrobials. To achieve this, CAMH broth containing different concentrations of antimicrobials and a bacterial inoculum at a cell density of 1 × 10^8^ CFU/mL was added to the microplates. The microplates were then incubated for 24 h at 25°C for the marine isolates and at 37°C for the reference strains.

### 2.3 Determination of Biofilm Formation Ability

The biofilm formation ability was determined by the crystal violet staining technique on 96‐well plates, using the protocol adapted from Drescher et al. (2019). The isolates were cultured at 25°C for 18 h in BHI broth, and then an aliquot of each one was transferred to the wells of the polystyrene plates in triplicates and incubated at 25°C for 48 h. After that, the wells were washed twice with phosphate buffered saline (PBS—NaCl, 8 g/L; KCl, 0.2 g/L; Na_2_HPO_4_, 1.44 g/L; and KH_2_PO^4^, 0.24 g/L) and stained with 0.1% crystal violet for 5 min. Subsequently, the biofilms were washed with 96% ethanol for 10 to 15 min, and the absorbance of the removed violet stain was evaluated using the SpectraMax microplate reader in the range of 190–570 nm.

The isolates were classified according to Stepanović et al. (2000) as: nonbiofilm forming (OD ≤ ODc), weak biofilm formers (ODc < OD ≤ 2xODc), moderate biofilm formers (2xODc < OD ≤ 4xODc) and strong biofilm formers (4xODc < OD), where ODc was the optical cutoff density determined as the mean OD plus three standard deviations from the negative control.

### 2.4 Antimicrobial Susceptibility Tests in Biofilm Condition

For the biofilm susceptibility test, we selected isolates susceptible to antimicrobials in planktonic conditions. To conduct the test, we used 96‐well plates with 200 μL of CAMH broth and 2 μL of McFarland 0.5 scale bacterial suspensions (1 × 10^8^ CFU/mL), which were then incubated at 25°C for 48 h. Afterward, the wells were washed twice with PBS and 200 μL of CAMH broth containing the different antimicrobial concentrations were added and incubated again at 25°C for 24 h. The antimicrobials used for biofilm tests were ciprofloxacin and tobramycin for *Pseudomonas* isolates, and ciprofloxacin and vancomycin for those of the Bacillales order. The concentrations used were based on the breakpoints for susceptibility of each antimicrobial in planktonic assays, as defined by EUCAST guidelines. Six concentrations were chosen above and two below these breakpoints. After this period, the minimum biofilm inhibitory concentration (MBIC) was visually determined, and the wells were again washed twice with PBS. Fresh CAMH broth was added to the wells and plates were then subjected to an ultrasound bath (35 kHz and 0,8 W/cm^2^) for 5 min. A 100 μL aliquot of each treatment was then serially diluted and drop‐plated (10 μL) on CAMH, and plates incubated at 25°C for 24 h. Afterward, the forming colonies were observed to verify the minimum biofilm eradication concentration (MBEC) of the antimicrobials (Ceri et al. 1999). Moreover, the noneradicated biofilms at the maximum concentrations applied had their forming colonies counted and CFU/mL estimated, which was compared to those from untreated biofilms.

### 2.5 Statistical Analysis

The parametric Student *t* test was used to compare the MIC and MBIC values (mean ± SD) of antimicrobials for the 15 bacterial isolates, and CFU/mL counts of noneradicated biofilms with their respective controls of 11 isolates. All parameters were measured in triplicates. *p* < 0.05 were considered statistically significant. GraphPad prism version 8.0.1 was used for data analysis.

## 3 Results

### 3.1 AMR Profile

The disk diffusion assay revealed that two *Paenibacillus* isolates (MET16 and MET17) were resistant to clindamycin, with MET17 also exhibiting resistance to erythromycin (Table 2). All other Bacillales isolates were susceptible to all antimicrobials tested. Among the *Pseudomonas* isolates, all six were resistant to aztreonam, and one isolate (MD330.9) was additionally resistant to ceftazidime.

**TABLE 2:** Antimicrobial susceptibility test result of the marine isolates that presented resistance to at least one antimicrobial.

Identification	Isolate	Antimicrobial	MIC (μg/mL)			
			*ATM*	*CAZ*	*ERY*	*CLIN*
*Pseudomonas* sp.	MD330.6	ATM	128	—	—	—
	MD330.9	ATM, CAZ	128	128	—	—
	MD330.10	ATM	> 2,048	—	—	—
	MD330.11	ATM	> 2048	—	—	—
	MD332.06	ATM	> 2048	—	—	—
	MD332.08	ATM	> 2048	—	—	—
*Paenibacillus* sp.	MET16	CLI	—	—	—	1
	MET17	CLI, ERY	—	—	1	2

*Note:* For the disk diffusion test, the antibiotics' acronyms to which the isolates were resistant are indicated. The minimum inhibitory concentrations (MIC) of these drugs are also indicated.

Abbreviations: ATM, aztreonam; CAZ, ceftazidime; CLI, clindamycin; ERY, erythromycin.

Broth microdilution confirmed these findings and provided quantitative MIC values. The MICs for erythromycin and clindamycin against the *Paenibacillus* isolates were 1 or 2 μg/mL. The MIC of ceftazidime for *Pseudomonas* MD330.9 was 128 μg/mL. For aztreonam, MICs of 128 μg/mL were determined for two *Pseudomonas* isolates (MD330.6 and MD330.9). The other four *Pseudomonas* isolates (MD330.10, MD330.11, MD332.06, MD332.08) exhibited MICs exceeding the maximum tested concentration of 2048 μg/mL (Table 2).

### 3.2 Biofilm Formation Ability

Biofilm formation capability varied among the 15 isolates. Six isolates were classified as nonbiofilm formers, two as weak, four as moderate and three as strong formers (Table 3). Taxonomic differences were observed: *Pseudomonas* isolates were distributed as nonformers (Braz et al. 2016), weak (Braz et al. 2016) or moderate (Braz et al. 2016) formers. In contrast, the Bacillales group included nonformers (Castaño‐Ortiz et al. 2023) and moderate formers (Braz et al. 2016), but no weak formers. Notably, this group contained three *Bacillus* isolates (MR39, MR46, MR53) that were strong biofilm formers.

**TABLE 3:** Classification of the intensity of biofilm formed by all 15 isolates, according to Stepanović et al. (2000)^b^.

Identification	Isolate	Average value of absorbance (570 nm)^a^	Classification^b^
*Pseudomonas* sp.	MD330.6	0.390	Weak
	MD330.9	0.154	Nonformer
	MD330.10	0.700	Moderate
	MD330.11	0.604	Moderate
	MD332.6	0.211	Nonformer
	MD332.8	0.425	Weak
Bacillales	MET16	0.140	Nonformer
	MET17	0.132	Nonformer
	MR6	0.625	Moderate
	MR16	0.202	Nonformer
	MR29	0.618	Moderate
	MR38	0.159	Nonformer
	MR39	0.771	Strong
	MR46	0.779	Strong
	MD53	1.133	Strong

^a^ Average value of biofilm triplicates.

^b^ Nonformers (OD ≤ ODc), weak formers (ODc < OD ≤ 2xODc), moderate formers (2xODc < OD ≤ 4xODc) and strong formers (4xODc < OD), where ODc is the optical cutoff density determined as the mean OD plus three standard deviations from the negative control.

### 3.3 Antimicrobial Susceptibility in Biofilm Condition

The susceptibility of preformed biofilms was tested. *Pseudomonas* biofilms were challenged with tobramycin and ciprofloxacin, while Bacillales biofilms were treated with ciprofloxacin and vancomycin, based on their planktonic susceptibility (Table 4).

**TABLE 4:** Values (in μg/mL) of minimal inhibitory concentration (MIC), minimal biofilm inhibitory concentration (MBIC) and minimal biofilm eradication concentration (MBEC) of ciprofloxacin, tobramycin and vancomycin for the bacterial isolates treated with these antimicrobials.

Identification	Isolate	Antimicrobials(μg/mL)								
		Ciprofloxacin			Tobramycin			Vancomycin		
		MIC	MBIC	MBEC	MIC	MBIC	MBEC	MIC	MBIC	MBEC
*Pseudomonas* sp.	MD330.6	0.062	> 128*	> 128	0.25	16*	32	NT	NT	NT
	MD330.9	0.062	1*	> 128	0.25	8*	> 32	NT	NT	NT
	MD330.10	0.031	> 128*	> 128	0.25	16*	> 32	NT	NT	NT
	MD330.11	0.062	> 128*	> 128	0.25	16*	> 32	NT	NT	NT
	MD332.6	0.062	> 128*	> 128	0.25	32*	> 32	NT	NT	NT
	MD332.8	0.25	> 128*	> 128	0.25	32*	> 32	NT	NT	NT
Bacillales	MET16	0.125	64*	128	NT	NT	NT	0.25	4*	> 64
	MET17	0.062	> 128*	> 128	NT	NT	NT	0.25	0.5*	> 64
	MR6	0.031	128*	128	NT	NT	NT	0.125	2*	> 64
	MR16	0.031	4*	> 128	NT	NT	NT	0.125	0.5*	> 64
	M29	0.031	4*	128	NT	NT	NT	0.125	4*	> 64
	MR38	0.031	16*	> 128	NT	NT	NT	1	1*	16
	MR39	0.062	8*	64	NT	NT	NT	0.125	4*	> 64
	MR46	0.031	< 0.5	64	NT	NT	NT	0.125	1*	8
	MR53	0.031	< 0.5	128	NT	NT	NT	0.125	0.5*	32

*Note:* Values proceeded by ‘<’ indicate that visual growth in biofilm condition (MBIC test) was not observed in the lowest concentration applied. Values proceeded by ‘>’ indicates that the maximum concentrations tested of these antimicrobials were not able to inhibit (MBIC) or eradicate (MBEC) biofilms. ‘*’ indicates significant differences between MIC and MBIC values (Student *t* test, *p* < 0.05); ‘NT’ is used when biofilms were not treated with the respective antimicrobial.

MBIC values were significantly higher than the corresponding planktonic MICs for the majority of isolates (93.3%, *p* < 0.05). Ciprofloxacin exhibited the greatest disparity, with six of the 15 biofilms showing no visible inhibition even at the maximum concentration tested. The MBICs for ciprofloxacin against *Bacillus* isolates MR46 and MR53 were below the lowest concentration tested and thus could not be determined or statistically compared.

Only one‐third of the biofilms (10 out of 30) were successfully eradicated. Among these, nine were from the Bacillales order and one was a *Pseudomonas* isolate (MD330.6). For Bacillales, ciprofloxacin was more effective, eradicating six biofilms compared to three for vancomycin. Only two *Bacillus* isolates (MR46 and MR53) were eradicated by both antimicrobials. The *Pseudomonas* isolate MD330.6 was eradicated only by the maximum concentration of tobramycin (32 μg/mL) but not by ciprofloxacin. For the remaining 20 biofilms (11 *Pseudomonas*, 9 *Bacillales*), MBEC could not be determined as eradication was not achieved at the maximum antimicrobial concentrations (Table 4).

**FIGURE 2:**
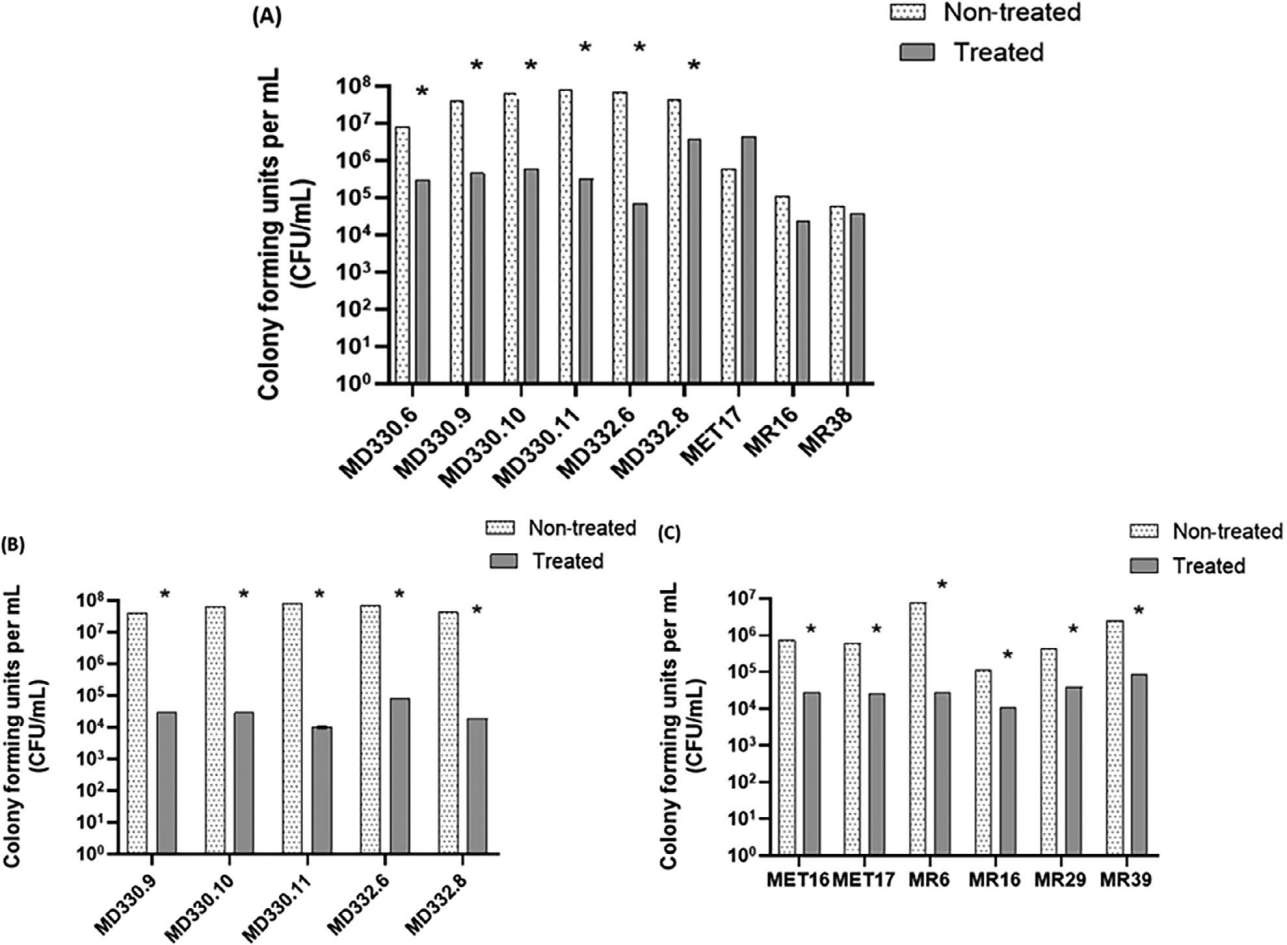
CFU/mL counts (mean ± SD) from isolates' biofilms that were noneradicated when treated at the maximum concentrations tested of: (A) ciprofloxacin; (B) tobramycin; (C) vancomycin. (*) indicates statistically significant differences between treated and nontreated biofilms (Student *t* test, *p* < 0.05).

Viable cell counts (CFU/mL) from noneradicated biofilms were compared to untreated controls (Figure 2). Significant reductions in viability were observed for 17 of the 20 noneradicated biofilms, with decreases ranging from 10^1^ to a maximum of 10^4^ CFU/mL. In contrast, biofilms of isolates MET17, MR16 and MR38 showed no significant reduction in CFU/mL when treated with ciprofloxacin, indicating a high level of biofilm‐specific tolerance to this antimicrobial.

## 4 Discussion

AMR is no longer confined to healthcare facilities, as natural environments can play a significant role in the acquisition and dissemination of resistance genes. In this context, this study aimed to evaluate the susceptibility to antibacterial drugs in both planktonic and biofilm conditions of bacterial isolates from deep marine sediment from the southern Brazilian coast. The results showed that eight isolates (six *Pseudomonas* sp. and two *Paenibacillus* sp.) showed resistance to at least one tested antimicrobial. All six *Pseudomonas* isolates showed a notable resistance profile, given the high MIC values observed for aztreonam. Bacterial high resistance to aztreonam is expected for *P. aeruginosa* strains from clinical origin, observed primarily due to mutations in operons related to the overexpression of the efflux pump (Jorth et al. 2017). Likewise, isolates of *Pseudomonas* from different environmental origins were also reported—in a much lower frequency—as resistant to this antimicrobial in previous studies (Luczkiewicz et al. 2015; Braz et al. 2016; Silverio et al. 2022). Nonetheless, nonspecific cellular mechanisms may also contribute to AMR in marine isolates.

Co‐selection of antimicrobial and metal resistance is observed in environmental bacteria. This occurs when genes related to metal resistance also confer cross‐resistance to antimicrobials, or when genetic markers for both types of resistance are located on the same mobile genetic element, such as plasmids (Pal et al. 2017; Squadrone 2020). In marine environments, the constant presence of metals may drive microorganisms to develop resistance strategies to deal with these elements (mainly efflux pump mechanisms) (Roberts 2011). One of our isolates, *Pseudomonas* MD330.6, had its genome analysed previously (NCBI BioProject PRJNA616366), in which resistant genes for mercury, copper, manganese and nickel were detected (Proenca et al. 2022). Among these, some genes of the *Mer* operon—that confer resistance to mercury—were present, which is a feature that has already been detected in antibiotic‐resistant marine isolates from genera *Pseudomonas*, *Bacillus* and *Pseudoalteromonas* (Dash et al. 2017), and *Bacillus thurigiensis* (Joshi et al. 2021), as they present this operon and antibiotic resistance genes co‐located on the same mobile genetic elements.

Regarding the resistance profile of the *Paenibacillus* MET16 and MET17, the AMR observed for lincosamides and macrolides has already been reported in the literature for this bacterial group (Bacillales order) in other oceanic regions (Barbosa et al. 2014; Dash et al. 2017; Joshi et al. 2021; Furlan et al. 2021). The double resistance seen in the MET17 is also expected, given their similar mechanism of action the clindamycin and erythromycin (Roberts 2011). Moreover, the prevalence of susceptibility among our Bacillales isolates may suggest the presence of susceptible genotypes in some bacterial groups within the RGC area. Despite significant environmental impact from highly navigation activities—from the south of Brazil (Patos Lagoon Estuary) and Uruguay (Plata River Basin)—and surrounding anthropogenic influences (da Silva et al. 2023), the spread of AMR does not seem to have manifested so far, at least in some Bacillales species from deep‐sea sediment.

In this context, this study makes a significant contribution to our understanding of environmental microbial resistance and tolerance by comparing MIC and MBIC values and evaluating MBIC and MBEC parameters for deep‐sea bacteria. Although research evaluating AMR in the environment has increased significantly, there are no studies evaluating the behaviour of these isolates in forms other than planktonic (Sweileh and Moh'd Mansour 2020). Therefore, comparison data about biofilms' tolerance to antimicrobials are made with clinical isolates or reference strains. In the literature, it has been reported that ciprofloxacin seems to be more efficient than tobramycin in eradicating clinical *P. aeruginosa* biofilms (Preston et al. 1996). This aspect, however, was different in our results, since the biofilms of these isolates tolerated the maximum concentration of ciprofloxacin, not being eradicated by this drug, whereas only one isolate (MD330.9) was successfully eradicated with tobramycin. This result shows a remarkable tolerance pattern for these isolates against two classes of antimicrobials—one fluoroquinolone and one aminoglycoside—under biofilm condition, which was distinct from the result they presented in the MIC test. Moreover, the biofilms from the Bacillales order were more sensitive to ciprofloxacin than those of *Pseudomonas* isolates. Only three Bacillales bacteria (MET17, MR16, MR38) were not eradicated by this antimicrobial. Nevertheless, we may highlight again the results of CFU/mL count from MET16 and MET17 biofilms, since they did not present significant differences from the untreated control, with slight (but not significant) increase of cell counts in MET17. Moreover, when Bacillales biofilms were treated with vancomycin, a higher number of isolates were not eradicated. Among these, it was possible to observe MET16 and MET17 again, indicating that the biofilms of our *Paenibacillus* isolates presented a high tolerance against two different classes of antimicrobials, one glycopeptide and one fluoroquinolone. Regarding reports related to tobramycin and vancomycin, they indicate that the effect of these antimicrobials could be time‐dependent, meaning that a longer exposure to these antimicrobials induces a more effective eradication of bacterial biofilms (Haagensen et al. 2017; Chen et al. 2020). So, it is possible that our MBEC data could be different with an exposure time higher than the 24 h employed. However, there is no established concept of what could be considered a prolonged exposure time for biofilms to antimicrobials, as it may vary greatly among studies (24 h up to 2 weeks) (Chen et al. 2020). Thus, from the same deep marine environment, we detected bacterial isolates from two distinct taxonomic groups that showed resistance and/or high tolerance in biofilm condition to different classes of antimicrobials.

In addition, our results corroborate data from the literature, as they clearly reinforce that bacterial cells tend to tolerate more harmful agents in biofilm than in their planktonic condition (Gilbert et al. 2002; Chen et al. 2020). Our data of MBIC (compared to respective MIC values) and MBEC demonstrate this tolerance, which may be related to different mechanisms, like the ability of their extracellular matrices to protect them against the antimicrobials tested (Gilbert et al. 2002). However, when relating the MBIC and MBEC values to the classification of biofilm formation using the crystal violet method, no alignment was detected between the results of biofilm intensity and the ability to tolerate antimicrobials. Some nonforming or weak biofilm‐forming isolates (e.g., MD330.9, MET17 and MR16) had their biofilms unaffected even at maximum antimicrobial concentrations, while other isolates classified as strong biofilm formers (e.g., MR46 and MR53) were eradicated with all antimicrobials tested, not necessarily at maximum concentration. The crystal violet staining method is commonly used to assess biofilm formation, but it may yield different biofilm intensity classifications due to factors such as manipulation methods, biofilm formation duration and extracellular matrix composition. These factors can affect dye penetration and lead to misinterpretation of results (Kragh et al. 2019). Therefore, the crystal violet assay is recommended as an initial screening tool for biofilms, which can be supplemented by more precise methods for further analysis, particularly when studying the impact of stressors (such as antimicrobials) on biofilms (Kragh et al. 2019).

Even if observed in a small number of isolates, these findings are significant as these bacterial features are not commonly reported in natural environments. The RGC region, besides being geologically characterised as a submarine fan, has been described as a methane cold seep site with methane hydrates occurrences (Miller et al. 2015). Due to global warming, this region faces the widespread dissociation of methane hydrates into gas, resulting in intense methane flux and massive methane releases through the sea sediment (Ketzer et al. 2020). This scenario creates a challenging and extreme environment for the microbial communities of sea sediment, from which our isolates come from. In this context, it is expected that these microorganisms present strategies—like tolerant biofilms—that may have been used to cope at least with these high methane concentrations.

Marine ecosystems face daily contamination from human activities. The antimicrobials disposal from different sectors, even under subinhibitory doses, can impact microbial communities (Nogales et al. 2011) and potentially select for resistant/tolerant bacteria. While resistant environmental bacteria may not belong to pathogenic species, they may serve as reservoirs of ARGs that may be transmitted to pathogens in the marine environment (Xu et al. 2023), posing not yet measured risks to human populations. As all isolates analysed in this study originated from deep marine sediments, our data contributes to understanding the selection and occurrence of resistant bacteria in these extreme natural environments that are part of the oceanic benthic realm, the least studied marine zone regarding microbial life (Laiolo et al. 2024). Furthermore, this study evaluated phenotypic characteristics of bacteria from a Brazilian oceanic site with a poorly described microbial diversity, offering unprecedented insights into deep‐sea bacteria, and shedding light on the impact of AMR and biofilm's tolerance to antimicrobials in marine environments. Mapping such phenotypic traits of these deep‐sea bacteria provides a baseline for understanding the environmental dimension of AMR, as these remote sediments may act as evolutionary reservoirs for ARGs. Such genes can potentially enter the global anthropogenic cycle through horizontal gene transfer or marine food webs, eventually impacting animal and human health, as well as the overall environment. Thus, these findings are relevant not only to understanding the conservation of marine microbial communities, but are also fundamental for ecosystems, animal and human wellness (Munn 2019), aligning with the principles of One Health and Global Health (Hernando‐Amado et al. 2019).

## Author Contributions

**Amanda Simão Dias:** conceptualization, writing – original draft, methodology, validation, formal analysis, investigation, writing – review and editing, data curation. **Sílvia Dias de Oliveira:** writing – review and editing, visualization, validation, conceptualization, resources, funding acquisition. **Renata Medina‐Silva:** funding acquisition, conceptualization, writing – review and editing, visualization, validation, project administration, data curation, supervision, resources.

## Funding

This work was supported by Petrobras (Brazil) (code 0050.0096017.15.9), Coordination of Superior Level Staff Improvement (CAPES, Brazil) (code 001) and the Brazilian National Council for Scientific and Technological Development (CNPq, Brazil) (R.M.‐S. and S.D.O. Research Career Award Scholarships).

## Ethics Statement

The authors have nothing to report.

## Conflicts of Interest

The authors declare no conflicts of interest.

## Data Availability

All data generated or analyzed during this study are included in this article.
